# Genetic Variability in the IGF-1 Axis Modulates Cancer-Associated Cachexia and Prognosis

**DOI:** 10.3390/cancers18111822

**Published:** 2026-06-02

**Authors:** Mariana Moreira Pires, Inês Guerra de Melo, Ana Carolina Leão Silva, Virgínia Rocha Dias, Cláudia Silva, Maria Paula Silva, Joana M. O. Santos, Tiago Ferreira, Valéria Tavares, Rui Medeiros

**Affiliations:** 1 Faculty of Medicine (FMUP), University of Porto, 4200-072 Porto, Portugal; marianamp006@gmail.com (M.M.P.); acleao.nutri@gmail.com (A.C.L.S.); 2 Molecular Oncology and Viral Pathology Group, IPO Porto Research Centre (CI-IPOP), Portuguese Oncology Institute of Porto (IPO Porto)/Department of Pathology and Laboratory Medicine/RISE—Associate Laboratory (Health Research Network)/Porto Comprehensive Cancer Centre Raquel Seruca (Porto.CCC), 4200-072 Porto, Portugal; ines.melo@ipoporto.min-saude.pt (I.G.d.M.); joana.oliveira.santos@ipoporto.min-saude.pt (J.M.O.S.); tiagoterras55@gmail.com (T.F.); valeria.tavares@ipoporto.min-saude.pt (V.T.); 3 Research Department, Portuguese League Against Cancer (NRNorte), 4200-172 Porto, Portugal; 4 Day Hospital, Portuguese Oncology Institute of Porto (IPO Porto), 4200-072 Porto, Portugal; virgi.dias@hotmail.com (V.R.D.); claudia_ssilva@hotmail.com (C.S.); 5 Palliative Care Service, Portuguese Oncology Institute of Porto (IPO Porto), 4200-072 Porto, Portugal; mpaulasilvajc@gmail.com; 6 Centre for the Research and Technology of Agro-Environmental and Biological Sciences (CITAB), University of Trás-os-Montes and Alto Douro, 5000-801 Vila Real, Portugal; 7 School of Medicine and Biomedical Sciences (EMCB), Fernando Pessoa University, 4420-096 Gondomar, Portugal; 8 Faculty of Health Sciences, Fernando Pessoa University, 4200-150 Porto, Portugal; 9 Instituto de Ciências Biomédicas Abel Salazar (ICBAS), University of Porto, 4050-313 Porto, Portugal; 10 European Cancer Organisation (ECO), 1040 Brussels, Belgium

**Keywords:** IGF-1, polymorphism, single-nucleotide, biomarkers, cachexia, neoplasms

## Abstract

**Background:** Cancer-associated cachexia (CAC) is a multifactorial syndrome driven by a profound metabolic and inflammatory dysregulation. Due to the central role of the insulin-like growth factor 1 (IGF-1) pathway in regulating muscle mass, energy metabolism, and inflammation, this study evaluated the relevance of five IGF-1 axis-related single-nucleotide polymorphisms (SNPs), namely *IGF1* rs6220, *insulin-like growth factor 1 receptor* (*IGF1R*) rs2016347 and rs2684788, *growth hormone receptor* (*GHR*) rs6873545, and *insulin receptor substrate 1* (*IRS1*) rs1801278. **Methods:** The impact of these variants on CAC onset and overall survival (OS) was assessed in a cohort of 140 cancer patients. **Results:** While overall-cohort analyses did not reach statistical significance, exploratory analyses suggested potential associations between the *IGF1* rs6220 GG and *GHR* rs6873545 CC genotypes and increased CAC risk in male patients. A trend for higher CAC prevalence was also noted in younger patients (<63 years) with the rs6873545 CC genotype. For pre-CAC and CAC patients, exploratory subgroup analyses on patients’ OS were conducted following no significant results in the overall cohort. Among older patients and those with high prognostic nutritional index (PNI; >44.2), the *IGF1* rs6220 G allele was associated with longer OS. Conversely, the *IGF1R* rs2016347 G allele and rs2684788 T allele were linked to poorer OS across multiple pre-CAC and CAC subgroups. The effects of *GHR* rs6873545 varied across subgroups, suggesting context-dependent activity. **Conclusions:** This study highlights the functional heterogeneity of IGF-1 axis-related genetic variants, indicating potential to serve as predictors of CAC. Given the exploratory nature of these findings, validation in larger cohorts is required to confirm the associations found.

## Simple Summary

Cancer-associated cachexia (CAC) is a multifactorial and paraneoplastic syndrome, characterised by systemic muscle wasting induced by chronic inflammation. This condition negatively affects patient prognosis through constant inflammation and metabolic dysregulation. Although no associations were observed in the overall cohort, post hoc exploratory analyses identified that *IGF1* rs6220 GG and *GHR* rs6873545 CC genotypes may increase CAC risk, particularly in male and younger patients. Furthermore, in pre-CAC and CAC patient subgroups, the *IGF1* rs6220 G allele was associated with longer overall survival (OS), while the minor alleles of the *IGF1R* variants were correlated with poor OS. Hence, the IGF-1 axis-related SNPs may serve as valuable non-invasive biomarkers for early CAC risk stratification and prognosis. Due to the exploratory nature of the results, they should be interpreted with caution and validated in larger and more consistent cohorts.

## 1. Introduction

Insulin-like growth factor 1 (IGF-1) is one of the most studied signalling molecules in the context of tumour metabolism, being responsible for cancer growth, metastasis, and resistance to cancer therapy. IGF-1, synthesised mainly in the liver and regulated by the growth hormone (GH), binds to the IGF-1 receptor (IGF1R), a transmembrane tyrosine kinase receptor. Upon activation, IGF1R stimulates RAS-RAF-mitogen-activated protein kinase (MAPK) and phosphoinositide 3-kinase (PI3K)-protein kinase B (Akt)-mechanistic target of rapamycin (mTOR) signalling pathways, thereby promoting cell proliferation, survival, and metabolic regulation [1]. Beyond its central role in the metabolic reprogramming of tumour cells, the IGF-1 axis also contributes to the onset and progression of cancer-related complications, such as cancer-associated cachexia (CAC) [2,3].

Cachexia is a common paraneoplastic syndrome and a life-threatening complication in cancer patients [4,5]. In this subpopulation, around 70% develop CAC, and over 20% of deaths are attributed to this condition [6]. CAC is defined by weight and muscle mass loss, accompanied by or without adipose tissue loss, that cannot be entirely reversed with nutritional support [7,8,9]. This multifactorial condition is characterised by a systemic pro- inflammatory state that plays a pivotal role in metabolic disarrangements that lead to disease pathogenesis. At the molecular level, CAC is marked by an imbalance between protein synthesis (anabolism) and protein degradation (catabolism), leading to impaired energy homeostasis and reduced muscle regenerative capacity [10,11,12]. The anabolic pathway is held by many molecular pathways, such as insulin/IGF-1-Akt-mTOR, MAPK, and SMAD 1/5/8, responsible for protein transcription leading to muscle growth [10,11,12]. In this setting, the pro-inflammatory cytokines (tumour necrosis factor-alpha (TNF-α), interleukin-1 (IL-1), -6 (IL-6), and -8 (IL-8), and interferon-gamma (IFNγ)) produced and released by cancer cells and the host immune system, drive muscle wasting by impairing regeneration and activating proteolytic pathways [8,13,14]. Skeletal muscle degradation occurs via the ubiquitin–proteasome system, the autophagy lysosomal pathway, and calpains, inducing a catabolic state [12,14,15]. Central to this process is the dysregulation of the PI3K-Akt-mTOR pathway, leading to Forkhead box O (FOXO)-mediated induction of muscle RING finger 1 (MuRF1) and Atrogin-1, accelerating proteolysis due to disruptions in the IGF-1 axis, which further contribute to CAC progression [11,12,14,15,16,17]. Compared with other metabolic factors, such as myostatin and pro-inflammatory cytokines, that promote energy imbalance and favour proteolysis, the IGF-1 axis represents an important target of research for understanding cachexia, due to its role in muscle mass metabolism [9,12,17].

The risk of developing CAC depends on a combination of patient, tumour, and treatment-related factors, including advanced age, male sex, poor nutritional or performance status, cancer type and stage, and treatment-related toxicities, particularly during the early phases of cancer management [13,18,19,20,21,22,23]. Notably, the body of evidence linking male sex to a higher CAC risk is still scarce [5,18,24].

Although there is no universal consensus on the diagnostic criteria for CAC, the Fearon criteria are the most extensively studied and widely referenced framework. To account for metabolic changes and the anorexia status, Fearon and co-workers, through an expert consensus, proposed a set of criteria to diagnose and classify the disease, including body mass index (BMI; <20 kg/m^2^) and progressive skeletal muscle mass loss (sarcopenia). These criteria classify CAC into three pathological stages: pre-CAC, CAC, and refractory CAC, with the latter typically occurring in advanced cancer patients with weak responsiveness to therapy [7,25,26]. Despite intensive research, the underlying molecular pathways remain poorly understood, and no predictive biomarkers have been successfully translated to clinical practice [5,24].

Genetic variants, particularly single-nucleotide polymorphisms (SNPs) arising from a single base substitution, may explain metabolic disarrangements and energetic imbalances in skeletal muscle and adipose tissue. Although most SNPs are functionally neutral, a subset can markedly affect gene expression and/or protein function, thereby influencing disease susceptibility [27]. In recent years, several SNPs modulating the activity of the IGF-1 signalling pathway have been identified, with the most studied including *IGF1* rs6220, *IGF1R* rs2016347 and rs2684788, *growth hormone receptor* (*GHR*) rs6873545, and *insulin receptor substrate 1* (*IRS1*) rs1801278 [28,29,30,31]. These genetic variants may serve as potential predictive biomarkers for the development of CAC. Hence, this study was designed to evaluate the relevance of these genetic variants to the occurrence of CAC and their impact on cancer patient survival.

## 2. Materials and Methods

### 2.1. Population Recruitment

A retrospective cohort study was conducted, enrolling cancer patients admitted to the Portuguese Oncology Institute of Porto (IPO Porto, Portugal), who were starting on or already undergoing first-line treatment or palliative care. The cohort included individuals of European descent aged 19 years or older, with an Eastern Cooperative Oncology Group Performance Status (ECOG-PS) of ≤3 and no restrictions regarding cancer type. Exclusion criteria comprised patients who requested a second medical opinion, were receiving medication for anorexia, had cognitive deficits, faced language barriers, or refused to participate in the study. Based on these criteria, a total of 140 cancer patients (Table 1) were consecutively recruited between March 2023 and May 2024, with a mean follow-up of 76.1 ± 4.0 weeks. The specific tumour types of the participants are described in Supplementary Table S1.

At the time of recruitment, data related to CAC status were collected by an experienced dietitian (A.C.L.S.) according to the Fearon criteria, considering unintentional weight loss over the previous six months, BMI, presence of anorexia, and systemic inflammation. Anthropometric measurements were also taken at the moment of recruitment to determine BMI (kg/m^2^), including weight using a digital scale (iHealth Nexus HS2S^®,^ iHealth Labs^®^, Sunnyvale, CA, USA) and height using a stadiometer. Body weight from the previous six months was self-reported by the patient. The presence of anorexia was determined using the Patient-Generated Subjective Global Assessment (PG-SGA), and systemic inflammation was assessed using the modified Glasgow Prognostic Score (mGPS ≥ 1), calculated from serum C-reactive protein (CRP) and albumin levels [32,33]. Following these criteria, cachectic patients were classified as having weight loss above 5% (last six months), or a BMI of 20 kg/m^2^ and weight loss > 2%, or muscle mass wasting and weight loss >2%. The pre-cachectic phase was defined by the presence of anorexia and weight loss ≤5%. In the other cases, patients were considered non-cachectic [7,34]. Upon assessment, 30% of patients were classified as cachectic, 11% as pre-cachectic, and the remaining patients as non-cachectic. Blood samples were collected during the same appointment, and the clinical history and demographic data were obtained from patients’ medical records.

To assess the patients’ nutritional and inflammation status, the prognostic nutritional index (PNI) and neutrophil-to-lymphocyte ratio (NLR) were also determined. The former has been recognised as a reliable predictor of postoperative complications across various malignancies, such as breast, liver, gastrointestinal, lung, ovary, and cervical cancers. Inclusively, lower PNI levels have been linked to poorer overall survival (OS) and disease-free survival [35,36]. PNI was calculated using the formula: serum albumin value (g/L) + 5 × total lymphocytes (×10^9^/L) [37]. Regarding NLR, defined as the ratio of absolute neutrophil count (×10^9^/L) to total lymphocyte count (×10^9^/L), it is a well-established biomarker of systemic inflammation [38]. Elevated NLR, reflecting increased neutrophils and decreased lymphocytes, suggests an impaired immune response to the tumour and has been linked to poor OS and advanced disease. Given its association with inflammation, NLR is particularly relevant in the context of CAC, where systemic inflammation is the principal driver of its development [39,40].

This study was approved by the Human Research Ethics Committee of IPO Porto (CES 131/022, approved on 28 July 2022), and all participants provided informed consent.

### 2.2. Sample Collection and DNA Extraction

Peripheral blood samples were collected from each patient at the time of recruitment via venipuncture. Samples were stored in tubes containing the anticoagulant ethylenediaminetetraacetic acid (EDTA) (BD Vacutainer Blood Collection Tube, Becton Dickinson, Franklin Lakes, NJ, USA).

Genomic DNA was isolated from the samples using the MagaBio Plus Virus DNA/RNA Purification Kit II (BSC71S1E, Bioflux^®^, Tokyo, Japan) with an automated extractor MGISP-NE32 (MGI Tech^®^, Guangdong, China), following the manufacturer’s instructions. The purity and concentration of the extracted DNA were assessed using a NanoDrop Lite spectrophotometer (Thermo Fisher Scientific, Waltham, MA, USA). Only DNA samples with an A260/A280 ratio close to 2.0 were deemed acceptable and stored at −20 °C until further use.

### 2.3. Polymorphism Selection and Genotyping

After a comprehensive review of the existing literature, polymorphism selection was conducted based on the following criteria: (i) SNPs in genes involved in the IGF-1 pathway; (ii) variants with functional impact on the activity of the encoded proteins and with previously described roles in cancer patients; (iii) variants linked to cachexia-associated metabolism; (iv) minor allele frequency (MAF) ≥10% to ensure sufficient representation of all SNPs’ genotypes in the study population; and (v) SNPs with available TaqMan^®^ genotyping assays (Supplementary Table S2). From the list, genetic variants with reported roles in CAC and/or cancer were prioritised. Subsequently, linkage disequilibrium (LD) between the genetic variants was considered to avoid overlapping effects. Applying these criteria, five SNPs were selected: *IGF1* rs6220, *IGF1R* rs2016347 and rs2684788, *GHR* rs6873545, and *IRS1* rs1801278 (Table 2). Further details on the functional consequence of each SNP are provided in the Supplementary Table S3.

Polymorphism genotyping was performed using a StepOnePlus^TM^ Real-Time Polymerase Chain Reaction (qPCR) system (Applied Biosystems^®^, Carlsbad, CA, USA) with a TaqMan probe-based strategy. Each PCR reaction contained a final volume of 6 μL, including 2.5 μL of TaqPath^TM^ ProAmp^TM^ Master Mix (1x) (Applied Biosystems^®^, Foster City, CA, USA), 2.375 μL of sterile water, 0.125 μL of pre-designed, validated TaqMan^®^ Genotyping assay (Applied Biosystems^®^, Foster City, CA, USA) for the respective SNP, and 1 μL of genomic DNA sample. The conditions of DNA amplification were previously described [41,42,43].

Two negative controls (lacking DNA) were included in each amplification reaction, and to further ensure the accuracy of SNP genotyping, 20% of the samples were tested in duplicate. Genotyping results were independently reviewed by three researchers, all blinded to patients’ clinicopathological data.

### 2.4. Statistical Analysis

Statistical analysis and graphical representations were executed using the Statistical Package for the Social Sciences (SPSS) software for Windows (version 30.0, IBM Corp., Armonk, NY, USA) and Microsoft Excel 365 (Microsoft Corporation, Redmond, WA, USA).

To evaluate genotype distribution, comparisons were made with Iberian population data reported in the Ensembl database. To assess deviations from expected genotype frequencies, the Hardy–Weinberg Equilibrium (HWE) was evaluated using the chi-square (χ^2^) test.

Data normality was examined through the Kolmogorov–Smirnov test. Given the exploratory nature of the study, continuous variables, including patients’ age and BMI, were dichotomised using the mean and median value, depending on whether they followed a normal or non-normal distribution, respectively. Hence, the cut-offs defined were derived from cohort distribution and do not represent biologic or clinical thresholds.

To enable analysis as nominal variables, the inflammatory indices PNI and NLR were stratified into binary categories of low and high. Patients were first grouped into tertiles based on the distribution of each index. These tertile-based groupings were then consolidated into two-level profiles. For PNI, values ≤44.2 (first tertile) were defined as low, while values in the second and third tertiles were considered high. Conversely, for NLR, values <3.6 (first and second tertiles) were classified as low, and those in the third tertile (≥3.6) as high.

Associations of the IGF-1 axis-related SNPs with CAC susceptibility and other patient characteristics (demographic and clinical pathological features) were examined employing the χ^2^ test or Fisher’s exact test, accordingly. For the significant associations, the odds ratio (OR) was determined whenever possible. In addition to the three CAC status subgroups (non-cachectic, pre-cachectic, and cachectic), two subgroups were taken into consideration by combining cachectic and pre-cachectic patients into a single pathologic entity (no CAC vs. pre-CAC + CAC).

The prognostic impact of IGF-1 axis-related SNPs was evaluated using Kaplan–Meier survival curves and the log-rank test or the Tarone–Ware test (depending on the proportional hazards assumption), considering the most suitable genetic model after an initial assessment of the survival curves. OS was defined as the interval between the date of patient recruitment (CAC diagnosis) and the date of death from any cause (event) or the last clinical assessment (censored). In addition to the entire cohort, post hoc exploratory analyses focusing on patients with pre-CAC and CAC were conducted, considering relevant patient characteristics. Owing to the relatively small sample size and the resulting constraints on statistical power, exploratory analyses by tumour type or treatment modality were not performed. Given the multitude of performed analyses, complete results are provided in Supplementary Tables, while significant exploratory findings are summarised in the main text.

The association between the genetic variants and mortality was also analysed using Cox proportional hazards models. Adjusted analyses incorporated key demographic and clinicopathological variables. Internal validation was carried out through bootstrap resampling with 1000 repetitions to assess the stability of the results.

All statistical tests were two-sided, and *p* < 0.05 was deemed statistically significant.

## 3. Results

### 3.1. Genotype Frequency of the SNPs

**Figure 1:**
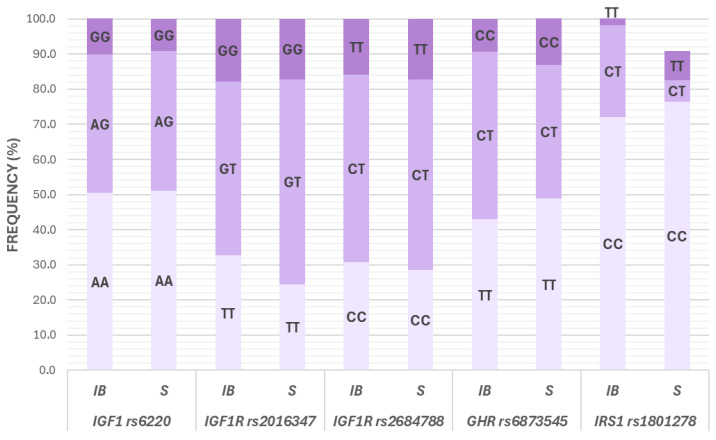
SNPs’ genotype distribution in the study cohort (S) compared with the reference Iberian population (IB).

Genotype distributions for each SNP are summarised in Figure 1. When compared to the expected frequencies in the Iberian population (https://www.ensembl.org, last accessed on 30 June 2025), the distribution of *IRS1* rs1801278 genotypes differed significantly (χ^2^, *p* < 0.05), indicating a deviation from HWE. Since a true association between this SNP and disease status cannot be assessed, and to prevent the implications of potential genotyping error and selection bias, *IRS1* rs1801278 was excluded from further statistical analyses. In opposition, the other variants conformed to HWE expectations (χ^2^ test, *p* > 0.05). Notably, no significant association was observed between the evaluated genetic variants and patients’ characteristics (χ^2^ test and Fisher’s exact test, *p* > 0.05).

### 3.2. Associations Between the SNPs and CAC Susceptibility: Entire Cohort and Post Hoc Exploratory Analyses

No significant associations between the evaluated SNPs and CAC susceptibility were observed in the overall cohort, irrespective of the genetic model applied (additive, recessive, or dominant). In contrast, relevant results were obtained in post hoc exploratory analyses according to patients’ sex and age, both recognised risk factors for cachexia [5,21]. Notably, for each SNP, multiple inheritance models (additive, dominant, and recessive) were evaluated, and analyses were performed using two Fearon classification approaches: (i) no CAC vs. pre-CAC vs. CAC and (ii) no CAC vs. pre-CAC + CAC. Given the large number of comparisons conducted, Table 3 summarises only statistically significant associations identified in these exploratory analyses (χ^2^ test or Fisher’s exact test, *p* < 0.05).

Sex-stratified analyses showed that male patients carrying the *IGF1* rs6220 GG genotype were significantly more prone to CAC than A allele carriers (Fisher’s exact test, *p* = 0.047; Table 3). Additionally, among male patients, the *GHR* rs6873545 CC genotype was observed exclusively in individuals with CAC (Fisher’s exact test, *p* < 0.05; Table 3).

In the exploratory analysis by patients’ age at recruitment (<63 vs. ≥63 years), among younger patients, those with CAC more frequently presented the *GHR* rs6873545 CC genotype (χ^2^ test, *p* = 0.027; Table 3).

No significant finding was observed for *IGF1R* rs2016347 and rs2684788 in the post hoc exploratory analyses according to patients’ sex and age (χ^2^ test or Fisher’s exact test, *p* > 0.05).

### 3.3. Associations Between the SNPs and Patients’ Overall Survival: Entire Cohort and Post Hoc Exploratory Analyses

**Figure 2:**
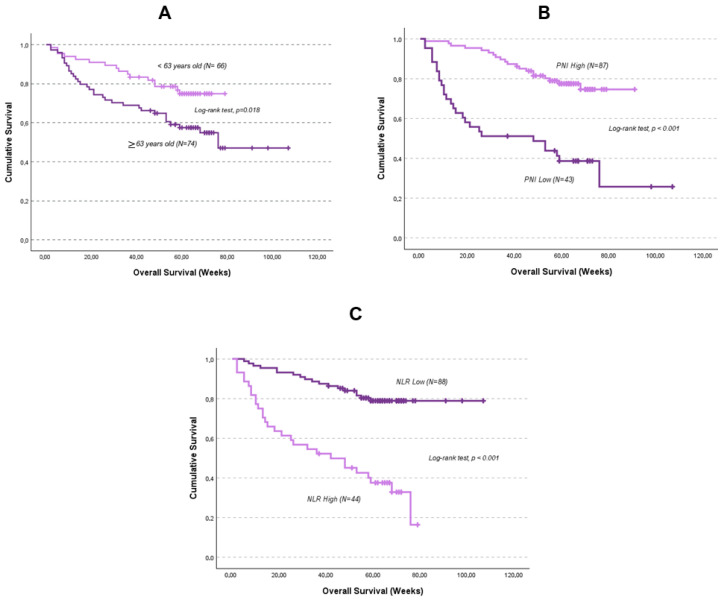
Overall survival (OS) by age, PNI, and NLR at recruitment. (**A**) Older patients had a lower survival than their counterparts (Log-rank test, *p* = 0.018). (**B**) Likewise, patients with lower PNI had a poor prognosis (Log-rank test, *p* < 0.001). (**C**) The same was observed for those with higher NLR (Log-rank test, *p* < 0.001).

Cachexia was shown to have a detrimental effect on patients’ survival (Log-rank test, *p* < 0.05). Namely, patients with either CAC or pre-CAC (N = 57) had a significantly lower survival than non-cachectic ones (mean OS of 47.77 ± 5.28 weeks and 95.07 ± 3.10 weeks, respectively; Log-rank test, *p* < 0.001). Consistently, affected patients showed an almost six-fold higher risk of death (hazard ratio (HR) = 5.78; 95% confidence interval (CI), 3.06–10.93; *p* < 0.001). Beyond CAC, other patient characteristics at recruitment significantly impacted their survival, namely age, PNI, and NLR (Figure 2A–C, respectively). While sex and BMI were not deemed predictors of survival in the cohort, these variables, together with patients’ age, PNI, and NLR at recruitment, were considered in exploratory analyses to assess the impact of the evaluated SNPs on patients’ prognosis, given their connection with CAC pathogenesis.

Considering the entire cohort for primary analyses, no significant associations between the evaluated SNPs and patient survival were detected, irrespective of the genetic model applied (additive, recessive, or dominant).

Focusing on patients with either pre-CAC or CAC, relevant associations were observed after post hoc exploratory analyses stratified by sex, age, BMI, PNI, or NLR (log-rank test or Tarone–Ware test; *p* < 0.05). The corresponding Cox regression analyses for each subgroup are presented in the Supplementary Table S4. For each clinical or demographic variable, all subgroup categories were evaluated, and additive, recessive, and dominant genetic models were tested for every SNP. Only statistically significant findings are reported.

**Figure 3:**
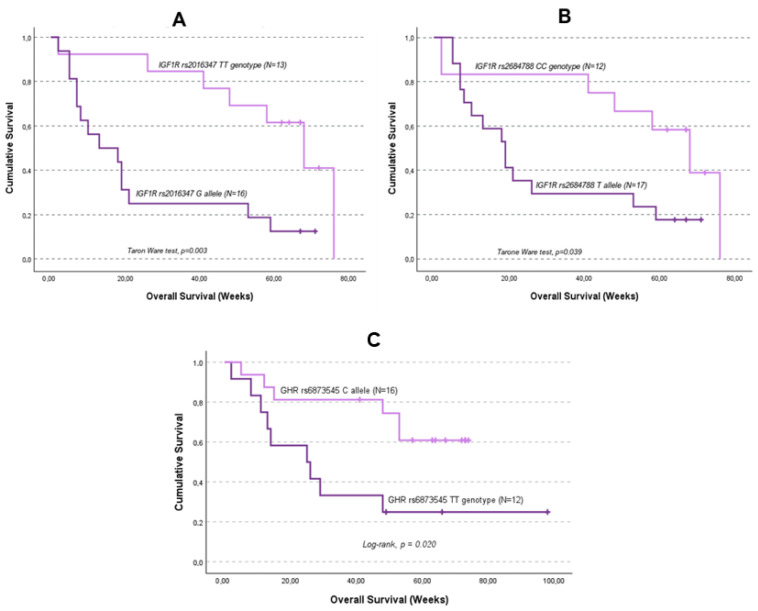
Impact of SNPs on the overall survival (OS) of pre-CAC and CAC patients classified by Fearon criteria and subdivided by sex. (**A**) Male patients with the *IGF1R* rs2016347 G allele had poorer OS than TT carriers (mean OS of 24.25 ± 5.91 weeks and 58.59 ± 6.82 weeks, respectively; Tarone–Ware test, *p* = 0.003). (**B**) In the same subgroup, the *IGF1R* rs2684788 T allele was linked to worse OS than the CC genotype (mean OS of 28.41 ± 5.98 weeks and 55.36 ± 8.28 weeks; Tarone–Ware test, *p* = 0.039). (**C**) On the other hand, female patients with the *GHR* rs6873545 C allele demonstrate a longer OS than TT genotype carriers (mean OS of 57.52 ± 6.10 weeks and 39.17 ± 10.34 weeks, respectively, Log-rank, *p* = 0.020). Among male patients, 9 were classified as pre-CAC and 20 as CAC, whereas among female patients, 6 were classified as pre-CAC and 22 as CAC.

The exploratory analysis by sex revealed that among male patients, the *IGF1R* rs2016347 G allele (GG/GT vs. TT, Tarone–Ware test, *p* = 0.003; Figure 3A) and the *IGF1R* rs2684788 T allele (CT/TT vs. CC, Tarone–Ware test, *p* = 0.039; Figure 3B) were associated with worse OS than their counterparts. In contrast, among female patients, those with the *GHR* rs6873545 C allele demonstrated longer survival time than the TT genotype carriers (CT/CC vs. TT, Log-rank test, *p* = 0.020; Figure 3C).

**Figure 4:**
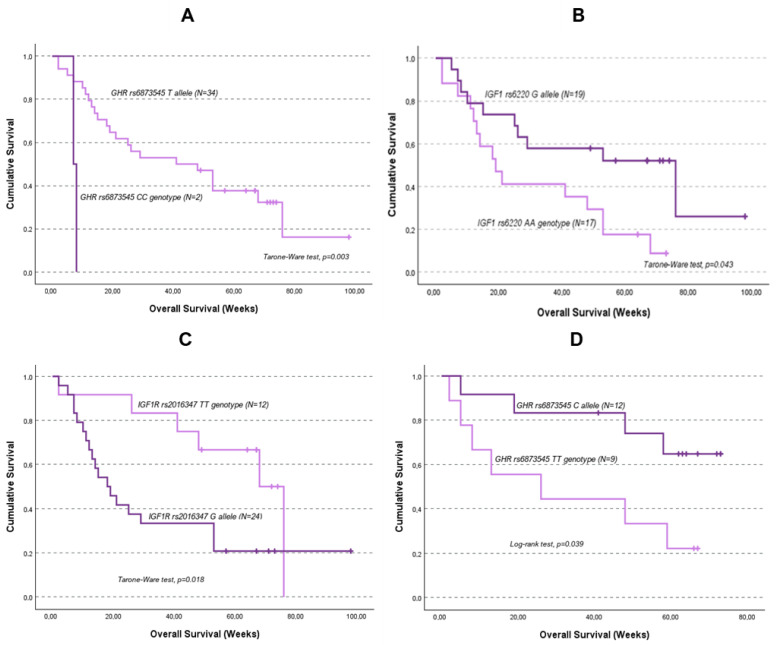
Impact of SNPs on the overall survival (OS) of pre-CAC and CAC patients subdivided by age. (**A**) Older patients carrying the *GHR* rs6873545 CC genotype had a worse prognosis than those with the T allele (mean OS of 7.50 ± 0.50 weeks and 46.07 ± 6.20 weeks, respectively; Tarone–Ware test, *p* = 0.003). (**B**) In contrast, the *IGF1* rs6220 G allele, in the same subgroup, was linked to a better OS compared to the counterparts (mean OS of 54.98 ± 9.00 weeks and 30.91 ± 5.92 weeks, respectively; Tarone–Ware test, *p* = 0.043). (**C**) The *IGF1R* rs2016347 G allele, in older patients, was associated with lower survival (mean OS of 36.04 ± 7.10 weeks and 59.08 ± 7.51 weeks, respectively; Tarone–Ware test, *p* = 0.018). (**D**) Among younger patients, those with the *GHR* rs6873545 C allele demonstrate a longer OS (mean OS of 59.13 ± 6.59 weeks and 32.78 ± 8.63 weeks, respectively; Log-rank test, *p* = 0.039). Among older patients, 11 were classified as pre-CAC and 25 as CAC, whereas among younger patients, 4 were classified as pre-CAC and 17 as CAC.

Moving on to the exploratory analysis according to patient age (Figure 4), the *GHR* rs6873545 CC genotype (Tarone–Ware test, *p* = 0.003; Figure 4A) and the *IGF1R* rs2016347 G allele (Tarone–Ware test, *p* = 0.018; Figure 4C) were associated with lower OS than their counterparts among older patients. In contrast, the *IGF1* rs6220 G allele was associated with better survival in the same group (Tarone–Ware test, *p* = 0.043; Figure 4B). Regarding the younger patients, carrying the *GHR* rs6873545 C allele was associated with a better prognosis (Log-rank test, *p* = 0.039; Figure 4D).

**Figure 5:**
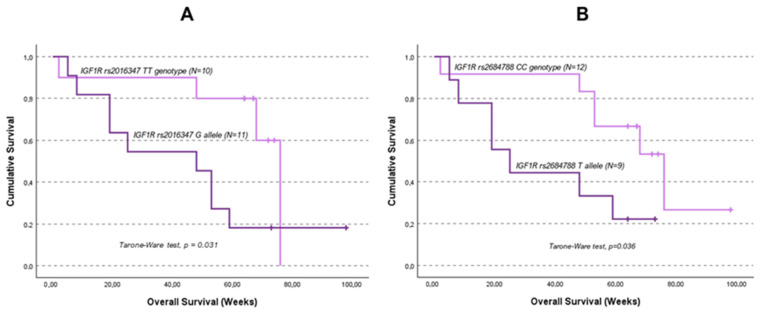
Impact of SNPs on the overall survival (OS) of pre-CAC and CAC patients classified by Fearon criteria and with elevated BMI. (**A**) The carriers of the *IGF1R* rs2016347 G allele demonstrate a poorer OS than those with the TT genotype (mean OS of 44.09 ± 9.39 weeks and 64.20 ± 8.24 weeks, respectively; Tarone–Ware test, *p* = 0.031). (**B**) The same effect was observed for the *IGF1R* rs2684788 T allele compared to CC genotype carriers (mean OS of 36.56 ± 8.50 weeks and 68.47 ± 8.45 weeks, respectively; Tarone–Ware test, *p* = 0.036). In the subgroup with elevated BMI, 8 were classified as pre-CAC and 13 as CAC.

In the exploratory analysis according to patient BMI, among patients with a high index (≥26 kg/m^2^), the *IGF1R* rs2016347 G allele (GT/GG vs. TT, Tarone–Ware test, *p* = 0.031; Figure 5A) and the *IGF1R* rs2684788 T allele (TC/TT vs. CC, Tarone–Ware test, *p* = 0.036; Figure 5B) were related to a poor OS.

**Figure 6:**
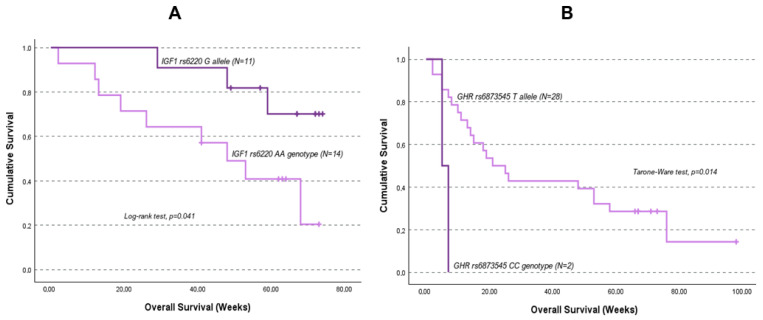
Impact of SNPs on the overall survival (OS) in pre-CAC and CAC patients classified by Fearon criteria and subdivided by PNI. (**A**) The *IGF1* rs6220 G allele in patients with higher PNI was associated with longer OS (mean OS of 65.79 ± 4.38 weeks and 45.09 ± 6.78 weeks, respectively; Log-rank test, *p* = 0.041). (**B**) The opposite effect was observed in patients with lower PNI carrying the *GHR* rs6873545 CC genotype when compared to their counterparts with the T allele (mean OS of 6.00 ± 1.00 weeks and 39.60 ± 6.70 weeks, respectively; Tarone–Ware test, *p* = 0.014). Among patients with higher PNI, 6 were classified as pre-CAC and 19 as CAC, whereas among those with lower PNI, 8 were classified as pre-CAC and 22 as CAC.

In the exploratory analysis according to patient PNI, among patients with high PNI (>44.2), those with the *IGF1* rs6220 G allele had an extended OS compared to AA genotype carriers (GA/GG vs. AA, Log-rank test, *p* = 0.041; Figure 6A). Regarding patients with low PNI (≤44.2), those carrying the *GHR* rs6873545 CC genotype exhibited less favourable survival outcomes than their counterparts (CC vs. CT/TT, Tarone–Ware test, *p* = 0.014; Figure 6B).

**Figure 7:**
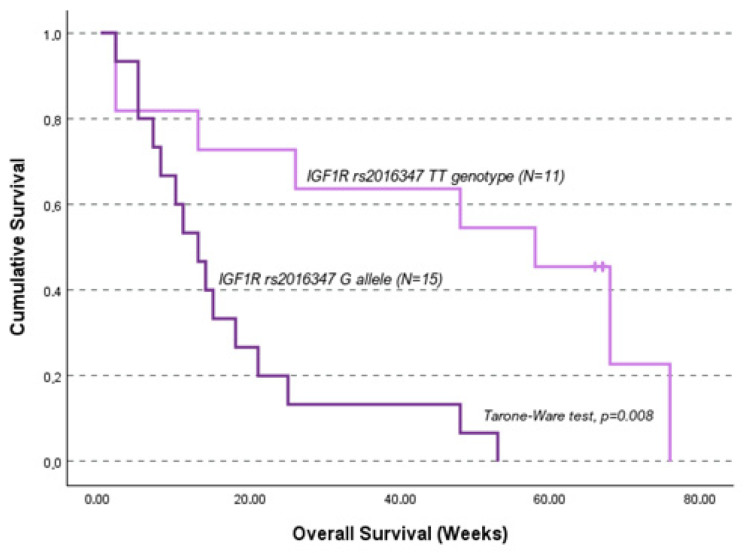
Overall survival (OS) of pre-CAC and CAC patients, classified by Fearon criteria, and with high NLR values, according to the *IGF1R* rs2016347. Carriers of the SNP G allele exhibited a lower OS than those with the TT genotype (mean OS of 17.00 ± 3.87 weeks and 46.27 ± 9.27 weeks, respectively; Tarone–Ware test, *p* = 0.008). In the subgroup with elevated NLR, 8 were classified as pre-CAC and 18 as CAC.

Lastly, in an exploratory analysis by patient NLR, patients with elevated NLR values showed significantly worse OS when carrying the *IGF1R* rs2016347 G allele compared with the TT genotype (GT/GG vs. TT; Tarone–Ware test, *p* = 0.008; Figure 7).

Focusing on patients with pre-CAC and CAC, a multivariable Cox proportional hazards model was performed to evaluate the independent prognostic value of the assessed SNPs. The analysis was adjusted for relevant patient characteristics identified in the Kaplan–Meier subgroup analyses of the genetic variants, including sex, age, BMI, PNI, and NLR at recruitment. Only SNPs and their corresponding genetic models that showed significant associations in the univariable analyses were included in the multivariable model. Given the exploratory and hypothesis-generating nature of the analysis, variable selection was performed using the backward Wald method. To assess the robustness and stability of the findings, bootstrap resampling with 1000 repetitions was conducted. A total of 35 death events were observed, corresponding to an events-per-variable (EPV) ratio of 11.7, indicating an acceptable balance between the number of events and the number of parameters included in the model [44]. The proportional hazards assumption was assessed using time-by-covariate interaction terms. This analysis suggested evidence of violation, indicating potential time-dependent effects. Hence, HRs should be interpreted with caution. Despite this limitation, the model was retained given its parsimonious structure, the acceptable EPV, and its exploratory objective. Based on the multivariable analysis, *IGF1* rs6220 and *IGF1R* rs2016347 emerged as the most relevant SNPs, both demonstrating independent prognostic value after adjustment for patient NLR (Table 4).

## 4. Discussion

Despite advances in cancer management and the growing implementation of personalised therapy strategies, disease recurrence rates remain alarmingly high, largely due to late-stage diagnosis, tumour aggressiveness, and acquired resistance to treatment [45]. These challenges not only worsen patient prognosis but also increase the likelihood of complications, such as CAC, which often limit therapeutic options to palliative care [46]. Emerging evidence suggests that alterations in cell metabolism contribute significantly to both malignancy and CAC [5]. In particular, chronic inflammation sustained by tumour cells and their microenvironment may serve as a key mechanistic bridge between cancer metabolic reprogramming and CAC development [13]. Namely, the dysregulation of the IGF-1 axis—a central player in metabolism, inflammation, and cellular survival—has been identified as a potential contributor to these processes [47]. To explore these associations and assess the role of the genetic component, five IGF-1 axis-related SNPs were analysed in this first study to evaluate the impact of these variants in an Iberian population in the CAC context.

Consistent with the literature, CAC had a detrimental impact on patients’ survival, which is in line with the growing body of evidence [5,9]. The SNPs were not significantly associated with patient characteristics or with cachexia status in the overall cohort. However, several associations of the SNPs with CAC status and patient survival emerged within specific subgroups, highlighting a potential role for IGF-1 axis genetics in the pathogenesis of CAC.

Starting with *IGF1* rs6220 (A > G), it is a genetic variant located on the 3′ untranslated region (UTR) of *IGF1*, thereby modulating IGF-1 expression. Specifically, the G allele has been described to increase IGF-1 levels [48]. While no association between the SNP and CAC status was detected in the overall cohort, the *IGF1* rs6220 GG genotype was linked to a higher risk of developing CAC among male patients, possibly due to increased IGF-1 levels that may trigger negative feedback that downregulates the PI3K-Akt-mTOR, allowing FOXO activity, inducing muscle wasting and atrophy [49]. Interestingly, when evaluating the effect on the survival of pre-CAC and CAC patients, it was observed that among older patients and those with high PNI, the SNP G allele was associated with longer OS. Consistent with these findings, the G allele was also shown to have a positive impact on the survival of pre-CAC and CAC patients in the multivariable analysis. Together, these results suggest that while the SNP GG genotype may have a pro-cachectic impact, the G allele could offer a positive influence on the survival of pre-CAC and CAC patients in specific clinical contexts. This apparent contradiction could be explained by the altered metabolic landscape in cachexia, where disruption of normal pathways allows compensatory signalling mechanisms, possibly involving residual anabolic activity, to modulate survival outcomes positively.

Concerning the *IGF1R* polymorphisms, rs2016347 (T > G) and rs2684788 (C > T), both variants are located in the 3′UTR and influence *IGF1R* expression and protein levels, modulating the circulating levels of IGF-1 [50,51,52]. The *IGF1R* rs2016347 G allele was associated with worse OS across multiple pre-CAC and CAC subgroups, including male patients, those at advanced age, those with high BMI, and those with elevated NLR. This negative impact of the SNP G allele was confirmed in the multivariable Cox analysis. Regarding the variant *IGF1R* rs2684788, among pre-CAC and CAC patients, the SNP T allele was also associated with poorer OS in male patients and those with high BMI. These findings suggest a consistent prognostic role of these *IGF1R* SNPs in pre-CAC and CAC patients. In line with observations in other malignancies, these variants may modulate the activity of the IGF-1 axis, influencing, via PI3K-Akt-mTOR signalling, the metabolic stability and the inflammation state underlying muscle wasting through IGF-1 resistance [49,53]. Considering the results and the existing literature, both the G allele of *IGF1R* rs2016347 and the T allele of *IGF1R* rs2684788 may exacerbate the severity of CAC, decreasing the compensatory anabolic responses that are crucial for preserving body mass, consequently negatively impacting patient survival [12,15,29].

As for *GHR* rs6873545 (T > C), among male patients, the SNP CC genotype was exclusively present among those with CAC, suggesting a pro-cachectic role. Regarding the SNP impact on patient prognosis, *GHR* rs6873545 demonstrated the most variable associations across pre-CAC and CAC subgroups. Namely, the C allele (CC and CT genotypes) was linked to improved survival in female and younger patients. In contrast, the CC genotype conferred a worse OS in older patients and those with low PNI. This effect was independent of the impact of heterozygous patients. These findings underscore the context-dependent nature of this SNP’s impact on survival and highlight the complexity of its role in cancer-associated outcomes. To the best of our knowledge, this is the first study that demonstrates the distinct impacts of the *GHR* rs6873545 C allele on the modulation of the IGF-1 axis across various subgroups of pre-CAC and CAC patients, when stratified by age and sex. According to the literature, the SNP C allele has been associated with enhanced GH signalling or responsiveness, which can modulate the anabolic responses and muscle degradation mechanisms, together with a continuous inflammatory state [54,55,56,57]. Inversely, the *GHR* rs6873545 T allele tags the full-length GHR isoform, which shows standard GH receptor expression and signalling [54]. Cachexia is well-known for the pro-inflammatory state promoted by the exacerbation from increased protein degradation over its synthesis pathways. The *GHR* rs6873545 C seems to increase the responsiveness to GH, which can induce a protective shield by partially enabling the restoration of IGF-1 levels and the respective IGF-1 signalling pathway [58]. In this context, the *d3-GHR* C allele helps improve and preserve body mass and attenuate the systemic inflammation status and metabolic exhaustion, particularly in female carriers who already have more capacity to resist this condition [21]. However, in older patients, the presence of this allele was consistently related to poor OS. Focusing on this finding, the *d3-GHR* C allele may modulate GHR expression by inhibiting the response to PI3K-AKT-mTOR signalling, thereby exacerbating catabolism and associated inflammation. The potential functional consequence of the *d3-GHR* C allele on the IGF-1 axis, the believed GHR-suppressed response, may make patients more vulnerable to other elderly-related complications that also worsen the capacity for survival.

Overall, the main key findings are that the *IGF1* rs6220 G allele may play a protective role in the clinical outcomes for pre-CAC and CAC patients, suggesting that IGF-1 enhances its expression and function, maintaining compensatory anabolic responses in the face of the catabolic inflammatory state and leading to preserving muscle mass and metabolic function. In contrast, the *IGF1R* rs2016347 G allele and rs2684788 T allele showed consistent results regarding the male subgroup and high BMI, suggesting these variants may be good predictors of patient prognosis, considering their context-dependent nature. These findings highlight the importance of functional heterogeneity among IGF-1 axis genetic variants and demonstrate genotype-specific modulation of CAC outcomes. Nonetheless, the *IGF1R* rs2016347 variant, like *IGF1* rs6220, was shown to have independent prognostic value, even after adjustment for patients’ inflammatory potential.

This study has limitations that should be considered when interpreting the findings. A primary limitation is the small sample size, which reduces statistical power to detect subtle associations, particularly in analyses of genetic variants with low MAF. The final Cox model was built on a relatively small subgroup, warranting caution in the interpretation of variable selection and model estimates. While bootstrap resampling was used to assess the model stability, the use of backward Wald selection may have increased the risk of overfitting in small samples. Therefore, these results should be interpreted as exploratory and hypothesis-generating rather than confirmatory. Regarding the methodology, the dichotomisation of continuous variables (for example, age, BMI, PNI, and NLR), while facilitating clinical interpretation, can limit the complexity of these exploratory findings, and the cut-offs should be considered provisional for hypothesis generating. Additionally, the dichotomisation may have reduced the statistical power. Notably, this approach was adopted to increase the clinical interpretation and reduce model complexity in a limited sample size. Consequently, the findings should be taken as exploratory and hypothesis-generating, requiring future validation with larger cohorts to validate these associations and assess the variables using continuous and more flexible strategies. Furthermore, PNI and NLR were determined in patients who may already have been undergoing anti-tumour treatments, thereby reflecting their inflammatory and nutritional status at the time of sample collection rather than a treatment naïve phase, which may limit our conclusions.

A relevant consideration of this study is that, although it is retrospective in nature, no tumour types were pre-selected or excluded. Hence, the cohort reflects the distribution of tumours diagnosed during the recruitment period, minimising potential bias toward specific tumour subtypes. However, it is important to acknowledge that our cohort did not include any cases of pancreatic ductal adenocarcinoma (PDAC), the tumour type most commonly associated with cachexia [59]. The absence of PDAC may have limited the generalisability of our findings to neoplasms with a higher predisposition for CAC. Nevertheless, other tumour types commonly associated with elevated cachexia risk were represented in the cohort, partially supporting the relevance of the findings across high-CAC malignancies. On the other hand, the heterogeneity of the cohort regarding tumour types may have limited the capacity to find tumour-specificity signatures. This inclusive approach was a pragmatic and exploratory decision to identify general biomarker signatures in a limited period of time, which can introduce confounding variables and explain distinct metabolic signatures. Due to limited sample size and the risk of misleading findings, multivariable adjustments were restricted to the most clinically relevant parameters, and the results were interpreted accordingly as exploratory and potentially time-dependent. Furthermore, because clinical characteristics and tumour types were unevenly distributed across cachexia status and patient subgroups, performing additional stratified analyses or adjusting to more models would bring underpowered estimates. Additionally, the evaluation was restricted to the Iberian population and its genetic diversity, highlighting the need for validation in larger, homogeneous, and prospective cohorts.

Last, this study did not account for potential gene–gene interactions. For example, research by Theron Niel et al. (2023) has shown that the interaction between *IGF1R* variants rs2016347 and rs2684788 may influence *IGF1R* gene expression at both the DNA and protein levels [29]. Taken together, the findings of the present study should be interpreted with caution, particularly the biological interpretation of each subgroup analysis should be reframed as hypothesis-generating rather than a conclusion. It is also important to note that circulating IGF-1 levels, gene expression, and downstream PI3K-Akt signalling activity were not evaluated, representing another limitation. Future studies incorporating these molecular and functional assessments would be essential to clarify the biological mechanisms underlying the observed associations, which remain largely speculative in the current literature.

Despite the limitations, the study offers meaningful insights consistent with previous evidence and underpinned by a biologically plausible rationale. The findings provide new insights into the complexity of CAC pathogenesis and new knowledge on the impact of IGF-1 axis-related SNPs in cancer patients, which is essential for identifying new biomarkers and targets for personalised disease management. Furthermore, this work underscores the importance of incorporating multifactorial risk markers to evaluate and stratify cancer patients who need metabolic assessment and intervention. Ultimately, these advancements could pave the way for more targeted and supportive interventions.

## 5. Conclusions

The present study investigated the implications of IGF-1 axis-related genetic variants for CAC susceptibility and prognosis, revealing that their impact is deeply modulated by biological context, such as sex and age. Despite the study limitations, these findings provide encouraging preliminary evidence that the IGF-1 axis may modulate both disease risk and survival of pre-CAC and CAC patients in a context-dependent manner. In summary, male carriers of the *IGF1* rs6220 GG and the *GHR* rs6873545 CC genotypes were at higher risk of developing CAC. The same was observed for patients aged 63 years or younger harbouring the rs6873545 CC genotype. Furthermore, the variants *IGF1* rs6220 and *IGF1R* rs2016347 emerged as independent predictors of death among individuals with pre-CAC and CAC. Overall, the impact of the evaluated SNPs seems to be context-dependent, suggesting their influence on prognosis may be modulated by complex interactions among biological and clinical factors. Due to the exploratory nature of these findings, they must be interpreted with caution, warranting post-validation. Future investigation should focus on validating these exploratory associations in larger and prospective cohorts to ensure higher statistical power. In addition, quantification of circulating IGF-1 levels in the study participants is necessary to evaluate the functional impact of these SNPs on gene expression and protein activity, as well as their downstream effects in the PI3K-AKT-mTOR signalling pathway. Once validated, these variants could be integrated with routine inflammatory and nutritional parameters to enhance predictive and prognostic models for CAC management. This integration would support targeted interventions and personalised decision-making, ultimately improving clinical outcomes for cancer patients.

## Figures and Tables

**Table 1:** Demographic, nutritional, and clinicopathological data of the study cohort (N = 140).

Variable		Mean ± SD	Frequency (%)
**Age (years)**	>63	63.08 ± 1.10	66 (47.1)
**Sex**	Males		64 (45.7)
	Females		76 (54.3)
**BMI** **(kg/m^2^)**	<26	26.02 ± 0.44	73 (52.1)
**PNI**	High		87 (62.1)
	Low		43 (30.7)
	Missing		10 (7.1)
**NLR**	Low		88 (62.9)
	High		44 (31.4)
	Missing		8 (5.7)
**ECOG-PS**	0		57 (40.7)
	1		39 (27.9)
	2		19 (13.6)
	3		3 (2.1)
	Missing		22 (15.7)
**Type of tumour**	Gastrointestinal		55 (39.3)
	Breast		18 (12.9)
	Lung		16 (11.4)
	Urologic		16 (11.4)
	Head and neck		12 (8.6)
	Gynaecological		9 (6.4)
	Skin		5 (3.6)
	Hematologic		5 (3.6)
	Sarcoma		3 (2.1)
	Central nervous system		1 (0.7)
**Metastatic disease**	Yes		90 (64.3)
**Other cancers**	Yes		8 (5.7)
**Cancer treatment**	Chemotherapy		43 (30.7)
	Palliative Support		32 (22.9)
	Surgery		15 (10.7)
	Immunotherapy		14 (10.0)
	Hormone Therapy		3 (2.1)
	Chemotherapy + Immunotherapy		18 (12.9)
	Chemotherapy + Radiotherapy		8 (5.7)
	Hormone therapy + Immunotherapy		5 (3.6)
	Chemotherapy + Hormone therapy		2 (1.4)

Abbreviations: Body Mass Index (BMI); Eastern Cooperative Oncology Group Performance Status (ECOG-PS); Prognostic Nutritional Index (PNI); Neutrophil-to-Lymphocyte Ratio (NLR); Standard deviation (SD).

**Table 2:** Selected SNPs to be evaluated in the study.

Gene	Location *	SNP (TaqMan^®^ Genotyping Assay)	Transition *	Functional Consequence *	MA: MAF in the Iberian Population *
*IGF1*	12q23.2	rs6220 (C___2801119_10)	A > G	3′UTR variant	G: 29.9%
*IGF1R*	15q26.3	rs2016347 (C___8723111_20)	T > G	3′UTR variant	G: 42.5%
		rs2684788 (C___1134378_30)	C > T	3′UTR variant	T: 42.5%
*GHR*	5p12	rs6873545 (C__28966089_10)	T > C	Intron variant	C: 33.2%
*IRS1*	2q36.3	rs1801278 (C___2384392_20)	C > T	Missense	T: 15.0%

* According to the Ensembl database (https://www.ensembl.org, last accessed on 30 June 2025). Abbreviations: Minor Allele (MA); Minor Allele Frequency (MAF); Single-Nucleotide Polymorphism (SNP); Untranslated Region (UTR).

**Table 3:** Distribution of *IGF1* rs6220 and *GHR* rs6873545 genotypes according to Fearon criteria in the male and younger patient subgroups.

Group	*IGF1* rs6220		*GHR* rs6873545					
	AA + AG N (%)	GG N (%)	TT N (%)	CT N (%)		CC N (%)	TT + CT N (%)	CC N (%)
**Male Patients**								
**No CAC**	35 (57.4)	0 (0.0)	15 (50.0)	20 (66.7)		0 (0)	35 (58.3)	0 (0)
**Pre-CAC**	9 (14.8)	0 (0.0)	6 (20.0)	3 (10.0)		0 (0)	9 (15.0)	0 (0)
**CAC**	17 (27.9)	3 (100.0)	9 (30.0)	7 (23.3)		4 (100)	16 (26.7)	4 (100)
***p*** **-Value**	0.047		0.037				0.015	
**No CAC**	-	-	15 (50.0)	20 (66.7)		0 (0)	35 (58.3)	0 (0)
**Pre-CAC + CAC**	-	-	15 (50.0)	10 (33.3)		4 (100)	25 (41.7)	4 (100)
***p*** **-Value**	-		0.027				0.037	
**OR** **(95% CI)**	-		-				2.40 (1.78–3.24)	
**Younger patients (≤63 years)**								
**No CAC**	-	-	-		-		43 (71.7)	2 (33.3)
**Pre-CAC**	-	-	-		-		4 (6.7)	0 (0.0)
**CAC**	-	-	-		-		13 (21.7)	4 (66.7)
***p*** **-Value**	-		-				0.027	

Abbreviations: Cancer-Associated Cachexia (CAC); Confidence Interval (CI); Number of Patients (N); Odds Ratio (OR).

**Table 4:** Multivariable Cox analysis on the risk of death among patients with CAC or pre-CAC (N = 55).

Variable	aHR (95% CI)	*p*-Value	Bootstrap *p*-Value
*IGF1* rs6220 (GG/AG vs. AA *)	0.47 (0.23–0.97)	0.040	0.02
*IGF1R* rs2016347(GG/TG vs. TT *)	4.20 (1.80–9.81)	<0.001	0.002
Patient NLR (high vs. low *)	5.94 (2.69–13.13)	<0.001	<0.001

* reference group. Abbreviations: Adjusted Hazard Ratio (aHR); Body Mass Index (BMI); Cancer-Associated Cachexia (CAC); Confidence Interval (CI); Neutrophil-to-Lymphocyte Ratio (NLR).

## Data Availability

The data presented in this study are available on request from the corresponding author.

## Supplementary Materials

The following supporting information can be downloaded at: https://www.mdpi.com/article/10.3390/cancers18111822/s1, Table S1: Tumour-type composition of the study cohort (N = 140); Table S2: TaqMan^®^ genotyping assay specifications and flanking sequences for selected SNPs; Table S3: Functional consequences of each IGF-1 axis-related SNP; Table S4: Summary of Kaplan-Meier exploratory analyses across clinical subgroups of pre-CAC and CAC patients, according to the evaluated IGF-1 axis-related SNPs.
