# Supplementary material for: Genetic Variability in the IGF-1 Axis Modulates Cancer-Associated Cachexia and Prognosis
Source: Cancers (Basel). 2026 Jun 2;18(11):1822. doi: 10.3390/cancers18111822 (PMC13256795; doi:10.3390/cancers18111822)
Supplement: Supplementary file 1 [file cancers-18-01822-s001.zip › cancers-4239825-supplementary.pdf]

cancers

MDPI

Genetic Variability in the IGF-1 Axis Modulates Cancer-Associated Cachexia and Prognosis

<u>**Supplementary Material**</u>

Supplementary Table S1: Tumour-type composition of the study cohort (N = 140).

Tumour type	Frequency (N)
Head and Neck (Unspecified)	12
Oesophageal	9
Oesophagogastric	1
Gastric	13
Colorectal	7
Colon	5
Rectal	7
Intestinal / Gastrointestinal tract	6
Liver	4
Biliary Tract	1
Gallbladder	1
Appendix	1
Pancreatic	0
Lung	16
Breast	18
Endometrial	2
Cervical	1
Uterine	1
Ovarian	4
Fallopian Tube	1
Prostate	8
Urothelial	3

Bladder	1
Kidney	4
Melanoma	4
Skin	1
Central Nervous System	1
Haematological (Unspecified)	3
Non-Hodgkin Lymphoma	1
Acute Myeloid Leukaemia	1
Angiosarcoma	1
Leiomyosarcoma	1
Liposarcoma	1
**Total**	140

https://doi.org/10.3390/cancers18111822

Supplementary Table S2: TaqMan® genotyping assay specifications and flanking sequences for selected SNPs.

Gene	SNP (TaqMan® genotyping assay)	MA: MAF in the Iberian population *	Flanking Sequence [VIC/FAM]
IGF1	**rs6220** (C___2801119_10)	G: 29.9 %	TAGAATATTATTTATAGTATTAAAC**[A/G]**AGGTTTTACTAGATAT GTAGTAACT
IGF1R	**rs2016347** (C___8723111_20)	G: 42.5 %	CGAATCCCCAGGGTAAAGGCGTGGG**[G/T]**CATTGGGTTTGCTCCC CTTGCTGCT
	**rs2684788** (C___1134378_30)	T: 42.5 %	CACTGTAGGTGACCCCTTGGAATAA**[C/T]**GGCCTCTCCTCTCGTG CACATACCT
GHR	**rs6873545** (C__28966089_10)	C: 33.2 %	GGGTCTAAGAAGCGTAAACATTGTG**[C/T]**CTTGTTGAAATACAGC CTCTAGGTA
IRS1	**rs1801278** (C___2384392_20)	T: 15.0 %	GTAGGCCTGCAAATGCTAGCAGCCC**[C/T]**GGGAGGTGCAGGGCC CAGTCTGCCC

* According to the Ensembl and Thermo Fisher database (https://www.ensembl.org. last accessed on 30th June 2025; https://www.thermofisher.com accessed on 12th May 2026). Abbreviations: minor allele (MA); minor allele frequency (MAF); single-nucleotide polymorphism (SNP).

Supplementary Table S3: Functional consequences of each IGF-1 axis-related SNP.

Gene	SNP	Alleles	MA: MAF in the Iberian population *	Functional consequence *	Reference
IGF1	rs6220	A>G	G: 29.9 %	**3′UTR variant**<br>The G allele increases IGF-1 expression and activity	[1]
IGF1R	rs2016347	T>G	G: 42.5 %	**3′UTR variant**<br>The T allele lowers *IGF1R* expression	[2]
	rs2684788	C>T	T: 42.5 %	**3′UTR variant**<br>Combined with the rs2016347 T allele leads to a reduction in *IGF1R* expression	[2]
GHR	rs6873545	T>C	C: 33.2 %	**Intron variant**<br>The C allele lowers IGF-1 plasmatic levels and promotes GH sensitivity	[3]
IRS1	rs1801278	C>T	T: 15.0 %	**Missense variant**<br>The T/A allele lowers IRS1 and PI3K-Akt activation	[4–6]

Abbreviations: 3' untranslated region (3'UTR); growth hormone receptor (GHR); insulin-like growth factor 1 (IGF1); insulin-like growth factor 1 receptor (IGF1R); insulin receptor substrate 1 (IRS1); minor allele frequency (MAF); phosphoinositide 3-kinase / protein kinase B (PI3K-Akt); single-nucleotide polymorphism (SNP).

https://doi.org/10.3390/cancers18111822

Supplementary Table S4: Summary of Kaplan-Meier exploratory analyses across clinical subgroups of pre-CAC and CAC patients, according to the evaluated IGF-1 axis-related SNPs.

**CAC Subgroup**		**SNP**	**Genotype/ Allele**	**N**	**Mean OS ± SE (weeks)**	***p-value***	**HR**	***p-value (Cox regression)***
**Sex**	Male	*IGF1R* rs2016347	TT	13	58.59 ± 6.82	**0.003**	1	**0.007**
			G	16	24.25 ± 5.90		3.83	
		IGF1R rs2684788	CC	12	55.36 ± 8.28	**0.039**	1	**0.047**
			T	17	28.41 ± 5.98		2.70	
	Female	*GHR* rs6873545	TT	12	39.17 ± 10.34	**0.020**	1	**0.028**
			C	16	57.52 ± 6.10		0.31	
**Age**	≥ 63 years	*IGF1R* rs2016347	TT	12	59.08 ± 7.51	**0.018**	1	**0.048**
			G	24	36.04 ± 7.09		2.55	
		IGF1 rs6220	AA	17	30.91 ± 5.92	**0.043**	1	**0.031**
			G	19	54.98 ± 9.00		0.40	
		GHR rs6873545	CC	2	7.50 ± 0.50	**0.003**	8.89	**0.012**
			T	34	46.07 ± 6.21		1	
	< 63 years	*GHR* rs6873545	TT	9	32.78 ± 8.63	**0.039**	1	0.055
			C	12	59.13 ± 6.59		0.30	
**BMI**	≥ 26 kg/m^2	*IGF1R* rs2016347	TT	10	64.20 ± 8.24	**0.031**	1	0.075
			G	11	44.09 ± 9.39		2.96	
		IGF1R rs2684788	CC	12	68.47 ± 8.45	**0.036**	1	0.056
			T	9	36.56 ± 8.50		3.14	
**PNI**	Hight	*IGF1* rs6220	AA	14	45.09 ± 6.78	**0.041**	1	0.057
			G	11	65.79 ± 4.38		0.28	
	Low	*GHR* rs6873545	CC	2	6.00 ± 1.00	**0.014**	NA	NA
			T	28	39.60 ± 6.70		NA	
**NLR**	High	*IGF1R* rs2016347	TT	11	46.27 ± 9.27	**0.008**	NA	NA
			G	15	17.00 ± 3.87		NA	

Abbreviations: body mass index (BMI); cancer-associated cachexia (CAC); hazard ratio (HR); neutrophil-to-lymphocyte ratio (NLR); Not applicable (NA); number of patients (N); overall survival (OS); prognostic nutritional index (PNI); standard deviation (SE); single-nucleotide polymorphism (SNP).

https://doi.org/10.3390/cancers18111822

References

1. Xu, G.-P., Chen, W.-X., Xie, W.-Y., & Wu, L.-F. (2018). The association between IGF1 Gene 3′-UTR polymorphisms and cancer risk. *Medicine*, *97*(51), e13829. https://doi.org/10.1097/MD.0000000000013829
2. Theron Niel. (2023). *A Mechanistic Evaluation of Naturally Occurring IGF1R 3′UTR Gene Variants Altering Disease Outcomes in Women*. Dominican University of California, San Rafael, CA. Retrieved from https://scholar.dominican.edu/biological-sciences-masters-theses/43
3. Marques, F. A., Lins, T. C., Lima, R. M., Fonseca, R. M. C., de França, N. M., de Oliveira, R. J., … Pogue, R. (2014). The exon 3 polymorphism of the growth hormone receptor is a severity-related factor for osteoporosis. *Endocrine*, *45*(3), 487–496. https://doi.org/10.1007/s12020-013-0004-1
4. Cheraghpour, M., Askari, M., Tierling, S., Shojaee, S., Sadeghi, A., Ketabi Moghadam, P., … Fatemi, N. (2023). A systematic review and meta-analysis for the association of the insulin-like growth factor1 pathway genetic polymorphisms with colorectal cancer susceptibility. *Frontiers in Oncology*, *13*(May), 1–20. https://doi.org/10.3389/fonc.2023.1168942
5. Li, P., Wang, L., Liu, L., Jiang, H., Ma, C., & Hao, T. (2014). Association between IRS-1 Gly972Arg polymorphism and colorectal cancer risk. *Tumor Biology*, *35*(7), 6581–6585. https://doi.org/10.1007/s13277-014-1900-6
6. Marín, C., Pérez-Martínez, P., Delgado-Lista, J., Gómez, P., Rodríguez, F., Yubero-Serrano, E. M., … López-Miranda, J. (2011). The insulin sensitivity response is determined by the interaction between the G972R polymorphism of the insulin receptor substrate 1 gene and dietary fat. *Molecular Nutrition & Food Research*, *55*(2), 328–335. https://doi.org/10.1002/mnfr.201000235

https://doi.org/10.3390/cancers18111822
